# Interplay between Penicillin-binding proteins and SEDS proteins promotes bacterial cell wall synthesis

**DOI:** 10.1038/srep43306

**Published:** 2017-02-24

**Authors:** Sophie Leclercq, Adeline Derouaux, Samir Olatunji, Claudine Fraipont, Alexander J. F. Egan, Waldemar Vollmer, Eefjan Breukink, Mohammed Terrak

**Affiliations:** 1 Centre d’Ingénierie des Protéines, University of Liège, B6a, Quartier Agora, allée du six Août 11, 4000 Liège 1, Belgium; 2 Institute for Cell and Molecular Biosciences, The Centre for Bacterial Cell Biology, Newcastle University, Richardson Road, Newcastle upon Tyne, NE2 4AX, UK; 3 Membrane Biochemistry and Biophysics, Department of Chemistry, Faculty of Science, Utrecht University, Padualaan 8, 3584 CH Utrecht, The Netherlands

## Abstract

Bacteria utilize specialized multi-protein machineries to synthesize the essential peptidoglycan (PG) cell wall during growth and division. The divisome controls septal PG synthesis and separation of daughter cells. In *E. coli*, the lipid II transporter candidate FtsW is thought to work in concert with the PG synthases penicillin-binding proteins PBP3 and PBP1b. Yet, the exact molecular mechanisms of their function in complexes are largely unknown. We show that FtsW interacts with PBP1b and lipid II and that PBP1b, FtsW and PBP3 co-purify suggesting that they form a trimeric complex. We also show that the large loop between transmembrane helices 7 and 8 of FtsW is important for the interaction with PBP3. Moreover, we found that FtsW, but not the other flippase candidate MurJ, impairs lipid II polymerization and peptide cross-linking activities of PBP1b, and that PBP3 relieves these inhibitory effects. All together the results suggest that FtsW interacts with lipid II preventing its polymerization by PBP1b unless PBP3 is also present, indicating that PBP3 facilitates lipid II release and/or its transfer to PBP1b after transport across the cytoplasmic membrane. This tight regulatory mechanism is consistent with the cell’s need to ensure appropriate use of the limited pool of lipid II.

Most bacteria surround their cytoplasmic membrane with a peptidoglycan (PG) sacculus which protects the cell from bursting due to the turgor and maintains cell shape. In order to propagate, bacteria have to enlarge and divide their cell envelope including their PG sacculus^1^. These processes are performed by specialized multiprotein complexes, the elongasome and divisome; each of them contains all the enzymatic activities required for the synthesis of new PG material and its insertion into the cell wall^2^. The substrate for PG synthesis is the precursor lipid II, which is synthesized on the inner face of the cytoplasmic membrane and subsequently translocated through the membrane by a flippase (FtsW and/or MurJ)^3^,^4^,^5^. Once on the periplasmic side of the membrane, lipid II is polymerized by the glycosyltransferase (GT) and the transpeptidase (TP, the target of penicillin) activities of the penicillin-binding proteins (PBPs)^6^,^7^ in coordination with interacting proteins of their respective networks, allowing growth and division of the bacterial cell^2^.

The divisome controls septal PG synthesis at mid-cell and its differentiation into the new poles of daughter cells. The components of the divisome span the cytoplasm, the cytoplasmic membrane, the periplasm and the outer membrane (in Gram-negative bacteria) and communicate by dynamic protein-protein interactions through these cell compartments. This organization allows the coordination between the cytoskeleton and the synthesis of precursors in the cytoplasm, their transport across the cytoplasmic membrane and septal peptidoglycan synthesis, and, in Gram-negative bacteria, the coordination with outer membrane invagination^8^,^9^. In *Escherichia coli*, the divisome includes over 20 proteins which assemble in an ordered and interdependent manner in two steps^10^; first, the tubulin-like FtsZ, ZipA, FtsA, ZapA-E and FtsEX localize at mid-cell underneath the cytoplasmic membrane. Later, the downstream components FtsK, FtsQ-FtsL-FtsB, FtsW-FtsI (PBP3) and FtsN join sequentially, some as preformed subcomplexes^11^,^12^, to form the mature divisome^13^. The bifunctional PG GTase/TPase PBP1b and its regulators (LpoB, CpoB, TolA) also associate with the divisome. PBP1b requires the PG TPase PBP3 which itself requires FtsW for septal localization^14^,^15^. PBP3 interacts *in vitro* with FtsW^12^, PBP1b^15^, and FtsN^16^ and with other proteins of the divisome^2^. Bacterial two-hybrid (BATH) analysis also revealed an interaction between PBP1b and FtsW^12^. FtsW is a polytopic membrane protein that belongs to the SEDS (shape, elongation, division, and sporulation) family and plays an essential role in cell division. It was shown to transport lipid II across membranes *in vitro*^3^. The inability of FtsW to transport bulky lipid II variants suggests the presence of a size-restricting pore that accommodates lipid II during transport^17^. Moreover, mutagenesis studies of FtsW showed that residues R145 and K153 of the transmembrane segment (TM) 4 are essential for flippase activity and the mutated proteins were incapable to rescue a conditional FtsW mutant strain^17^. The periplasmic loop (E240-A249) between transmembrane helices (TMs) 7 and 8 is essential for septal PG assembly and the loop between TMs 9 and 10 is required for the localization of PBP3 at the septum^18^.

A second candidate protein proposed to flip lipid II, MurJ, belongs to the multidrug/oligosaccharidyl-lipid/polysaccharide (MOP) exporter superfamily^4^,^19^. MurJ is essential for viability and peptidoglycan synthesis in *E. coli.* The heterologous expression of the O-antigen flippase Wzk from *Helicobacter pylori* in *E. coli* was able to complement the loss of MurJ^20^. A recently proposed third flippase candidate, Amj of *Bacillus subtilis*, is the founding member of new membrane protein family^21^. It was recently reported that the SEDS protein RodA is essential for viability of a *B. subtilis* mutant lacking all class A PBPs, and has a GTase activity^22^. Hence, different roles for these different families of integral membrane proteins involved with PG growth have been proposed, showing the need to clarify their precise biochemical activities.

Several PG enzymes are known to engage in functional interactions with divisome proteins. PBP1b is stimulated by FtsN and the outer membrane (OM) lipoprotein LpoB, which binds to its UB2H domain^16^,^23^,^24^,^25^, and the dynamic interactions between LpoB, CpoB and TolA with PBP1b facilitate the coordination between PG synthesis and OM invagination^8^. FtsQ, FtsL and FtsB associate in a stable ternary complex^11^ but their exact roles are unknown. FtsN is the last protein to localize to the division site and interacts with PBP1b, PBP3, FtsQ and FtsW^2^,^16^. It is believed to play an important role in the stability of the divisome and the initiation of the cell envelope constriction by activating septal PG synthesis through its interactions with FtsA and FtsQLB^26^,^27^. Large complexes containing more than two divisome proteins have been isolated in a few cases, the largest being a 1 MDa complex containing FtsZ, ZipA, FtsK, FtsQLB, and FtsN from *E. coli*^28^. In another study the proteins DivIB, DivIC, FtsL, PBP2x and FtsW from *Streptococcus pneumoniae* were co-expressed in *E. coli* and complexes composed of up to five proteins were isolated^29^.

While it is now evident that the PG synthase activities are regulated within the divisome and coordinated with the progression of the cell cycle through protein-protein interactions, the molecular mechanisms of these processes remain largely unknown. The *E. coli* septum PG synthesis components of the divisome includes FtsQ-FtsL-FtsB, FtsW-PBP3, PBP1b, FtsN, LpoB, CpoB and TolA (Fig. 1). In this work, we have investigated the divisome enzymes directly involved in septum synthesis, the synthases PBP1b and PBP3 and the lipid II flippase candidate FtsW, with the aim to understand how the interactions between them regulate the PG synthesis activities.

## Results

### FtsW, PBP3 and PBP1b form a ternary complex *in vitro*

**Figure 2:**
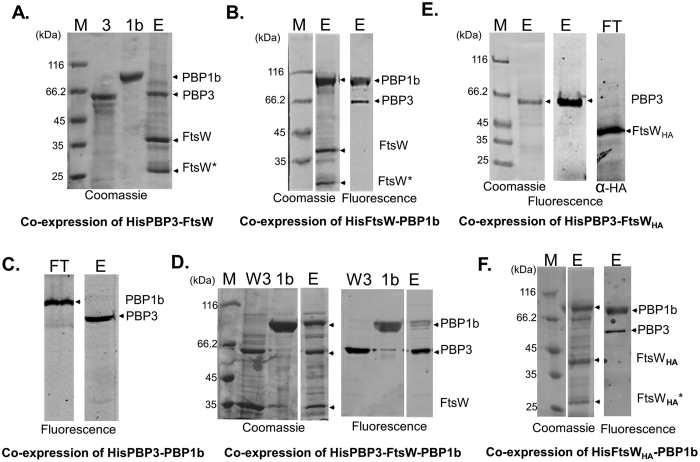
Interactions between FtsW, PBP3 and PBP1b. The proteins were co-expressed in *E. coli* cells; one contains a His-Tag and was used as bait while the other proteins are untagged. The co-expressed proteins are indicated below each panel. The expressed protein cannot be detected in the total extract but only after affinity purification. The cytoplasmic membranes from cells expressing the indicated proteins were solubilized with detergent followed by a purification step over a Ni-affinity column. The eluted fractions or flow-through fractions were labeled by fluorescent ampicillin and analyzed by SDS-PAGE. The gels were first subjected to fluorescence imaging followed by Coomassie blue staining. 3, 1b and W3 indicate lanes with purified PBP3, PBP1b and FtsW-PBP3, respectively (size controls). Lanes E depict elution fractions from the Ni-affinity columns and FT the flow-through. M, protein standards. The bands of PBP1b, PBP3 and FtsW are indicated on the right side of the gels. FtsW* indicates an FtsW degradation product. In panel E, the flow-through fraction (FT) was analyzed by immunoblotting using antibodies against the epitope HA to detect FtsW_HA_.

As a transporter candidate, FtsW is probably the last protein to interact with lipid II before its polymerization by the glycosyltransferase activity of a bifunctional PBP. PBP1b localizes to the septum and interacts with several divisome proteins and thus, it is likely to be the first enzyme to process lipid II after its translocation during cell division. PBP3 forms a complex with FtsW and its specific transpeptidase activity is essential for cell division. Binary interactions between FtsW, PBP3 and PBP1b have been shown by the bacterial two hybrids (BATH) system^15^,^30^,^31^ and confirmed *in vitro*^12^,^15^ except for the interaction between PBP1b and FtsW that was only shown by BATH^12^. We sought to complete the characterization of this protein triplet by analyzing the interaction between PBP1b and FtsW *in vitro* and to isolate the ternary complex. The proteins were co-expressed in *E. coli* followed by affinity co-purification with one of the two proteins containing an N-terminal His-tag (bait) and the second one left untagged (prey). It is noteworthy that the latter method was found to be more efficient in the detection of complexes than the purification of the separate proteins followed by reconstitution *in vitro*. This might be due to the fact that once a sufficiently stable complex is formed it can be solubilized without dissociating while, when the proteins are solubilized separately, the detergent might interfere with some of the interactions. The co-expression system was first tested using PBP3 and FtsW that were previously shown to form a sub-complex^12^. After co-expression and membrane solubilization with detergent (DDM) the proteins were loaded on a Nickel affinity column followed by washing steps and imidazole elution. The fractions were then analyzed using SDS-PAGE and fluorescent-ampicillin to label the PBPs. As expected, the results show that PBP3 and FtsW co-eluted (Fig. 2A), confirming their interaction. The same strategy was then applied to the HisFtsW-PBP1b and HisPBP3-PBP1b pairs. We found that HisFtsW and PBP1b co-eluted (Fig. 2B). However, in the case of HisPBP3-PBP1b only tagged PBP3 was recovered after elution, and PBP1b was found in the flow through (Fig. 2C). These results show, firstly, that untagged PBP1b does not bind nonspecifically to the column in the absence of interacting partner and therefore, that FtsW and PBP1b interact specifically. Secondly, the absence of detectable interaction between PBP1b and PBP3 by this method is probably due to weak and/or dynamic interaction between the two proteins. This conclusion is consistent with previous results showing that co-immunoprecipitation of PBP1b and PBP3 occurs after cross-linking^15^. Consequently, we conclude that the FtsW-PBP3 and FtsW-PBP1b interactions are stronger or less dynamic than the PBP1b-PBP3 interaction. Interestingly, labelling of the PBPs with fluorescent ampicillin after elution revealed that the native *E. coli* PBP3 co-purified with HisFtsW-PBP1b (Fig. 2B). This result was confirmed by the co-expression and co-purification of HisPBP3, FtsW and PBP1b, which confirmed the co-elution of the three proteins (Fig. 2D). Since PBP1b was not retained with HisPBP3 but only when FtsW was co-expressed, this suggests that PBP1b is bound to the ternary complex via FtsW. The construct of PBP1b also contained FtsN but this protein did not co-elute with the three proteins, suggesting that its interactions with the ternary complex is weak.

### The loop between TM 7 and 8 of FtsW is essential for the formation of the FtsW-PBP3 complex

The first 56 N-terminal amino acids of PBP3, containing the transmembrane (TM) segment, are essential for its localization and for the interaction with the TMs of FtsW^32^,^33^. A model of the interaction between the TMs of FtsW and PBP3 was proposed using a co-evolutionary approach^34^. In addition, the loop between TM9 and 10 of FtsW is involved in the interaction with PBP3 and PBP1b^12^,^18^. Mutagenesis studies in the large loop between TM7 and 8 implied its importance for the function of FtsW, but the exact role was not determined^18^,^35^. The insertion of the 9 amino acid hemagglutinin (HA) peptide in the TM7/8 loop between residues 293–294 of FtsW yields a functional protein (FtsW_HA_) as shown by *in vivo* complementation^18^. However, when FtsW_HA_ was co-expressed with HisPBP3, only tagged PBP3 was recovered during the elution step (Fig. 2E) (under the same conditions, wild-type FtsW forms a stable complex with PBP3 (Fig. 2A)). This indicates that the binding between the two proteins was affected by the insertion of the HA peptide and that the TM7/8 loop is crucial for a strong interaction with PBP3. Since the PBP3-PBP1b and PBP3-FtsW_HA_ interactions are too weak to observe pairwise complexes using the co-expression/co-purification method, we hypothesized that the simultaneous interaction of PBP3 with PBP1b and FtsW_HA_ might stabilize a ternary complex. Indeed, when HisFtsW_HA_ and PBP1b were co-expressed and co-purified, the two proteins co-eluted with PBP3 (Fig. 2F), supporting our hypothesis and confirming the presence of a ternary complex and not of two subpopulations of FtsW-PBP1b and FtsW-PBP3 complexes. To our knowledge, this is the first time that such a complex was isolated, suggesting that the transport of the lipid II precursor is coupled with its polymerization and insertion into the cell wall.

### FtsW inhibits lipid II polymerization by PBP1b if not in complex with PBP3

**Figure 3:**
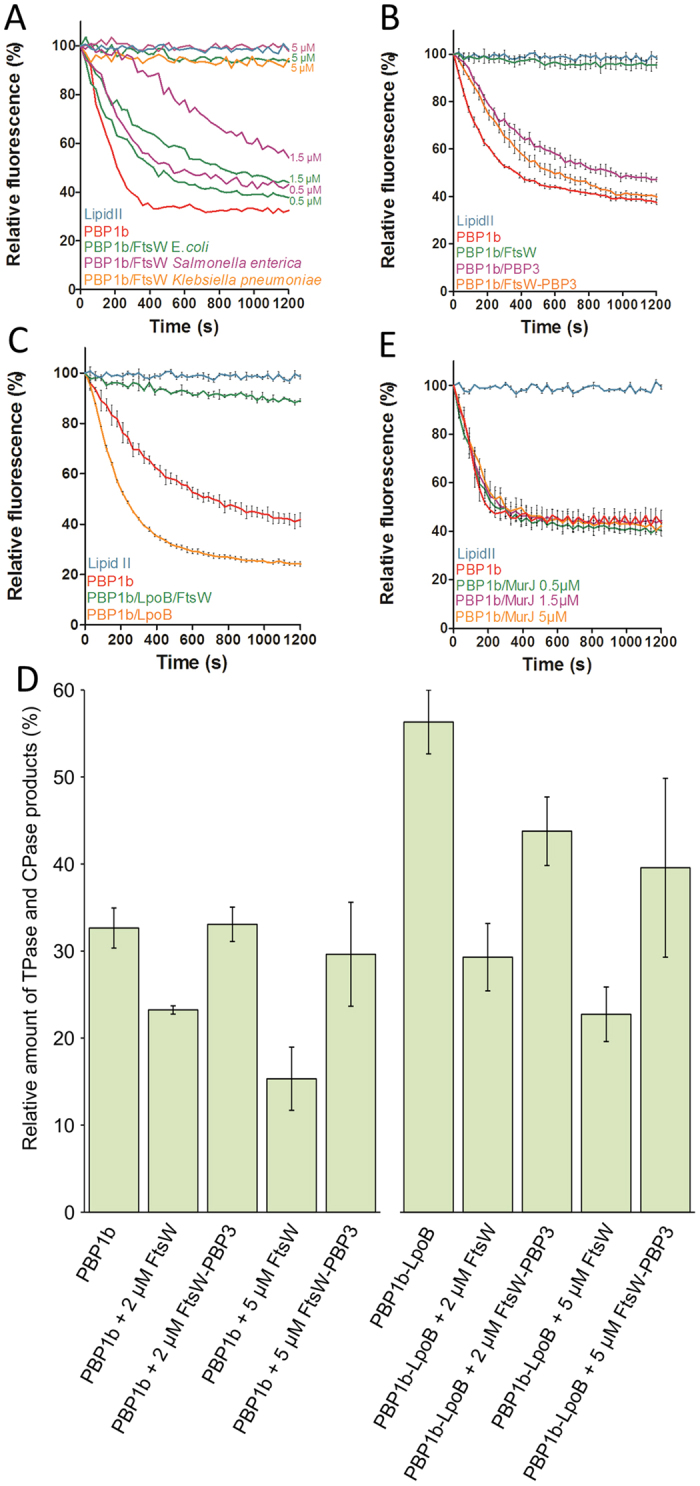
Effect of FtsW, PBP3 and the FtsW-PBP3 complex on the activities of PBP1b. (**A**–**C**) Continuous fluorescence assay to measure the polymerization (GT activity) with dansyl-lipid II. The fluorescence decreases over time upon polymerization of dansyl-lipid II by PBP1b. A. inhibition of lipid II polymerization by FtsW from *E. coli, S. enterica* and *K. pneumoniae*. (**B**) In contrast to FtsW (5 μM), the polymerase activity of PBP1b is not inhibited by the FtsW-PBP3 complex (5 μM) and slightly decreases in the presence of PBP3 (5 μM). (**C**) PBP1b activation by LpoB (200 nM) does not suppress the inhibitory effect of FtsW (5 μM). (**D**) Effect of FtsW and the FtsW-PBP3 complex on TPase activity of PBP1b. The percentage of muropeptide products detected by HPLC analysis of PG produced *in vitro* by PBP1b in the presence of the proteins indicated beneath each bar (endpoint assay). Total TPase activity is the sum of activities derived from the TPase domain of PBP1b, including peptide cross-linking and carboxypeptidase activities. Values are the mean ± SD of three experiments. Examples of the corresponding HPLC chromatograms are depicted in Supplemental Figure S1. The effects of FtsW and FtsW-PBP3 on PBP1b’s PBP domain parallel the effects on GTase in A, consistent with the coupling of both domains. E. In contrast to FtsW, MurJ (0.5–5 μM) has no effect on the polymerase activity of PBP1b.

In *E. coli*, the complex formed by PBP1b, PBP3 and FtsW constitutes the core-enzyme of the divisome that interacts with the lipid II substrate. In order to understand the effect of the interactions between these proteins on lipid II polymerization we analyzed the activity of PBP1b alone and in the presence of either FtsW or PBP3, or of the complex FtsW-PBP3. The glycosyltransferase activity of *E. coli* PBP1b was monitored by the continuous fluorescence method using dansyl lipid II as substrate^36^,^37^. The polymerase reaction is accompanied by a decrease in the fluorescence signal as a function of time. However, when FtsW from *E. coli, Klebsiella pneumoniae* or *Salmonella enterica* were added to the reaction mixture, the GT activity of PBP1b was inhibited in a concentration-dependent manner (Fig. 3A). Under the same conditions, PBP3 from *E. coli* slightly lowered the activity of PBP1b (Fig. 3B). Remarkably, the inhibitory effect of FtsW was suppressed in the presence of the co-purified *E. coli* FtsW-PBP3 complex and the activity of PBP1b was restored to a level close to that of PBP1b alone (Fig. 3B). FtsW inhibition could be the result of direct interaction with PBP1b or lipid II as FtsW potentially interacts with both.

The lipoprotein LpoB interacts with PBP1b and stimulates its activity^23^,^24^,^25^. In order to test whether PBP1b activation by LpoB could reverse the inhibitory effect of FtsW on lipid II polymerization by PBP1b, the experiment was performed in the presence of LpoB. As previously published, LpoB activated the GT activity of PBP1b. FtsW was still able to inhibit PBP1b in the presence of LpoB (Fig. 3C).

The GT and TPase activity of class A PBPs is coupled^38^,^39^, and under certain conditions the stimulated PBP domain can perform carboxypeptidase (CPase) reactions^40^, thus we sought to test whether the inhibition of GTase activity by FtsW leads to a concomitant decrease in TPase/CPase activity by analyzing the composition of the PG produced. Indeed, after a 2 h incubation period with radioactive lipid II the total TPase/CPase activity of PBP1b was reduced by FtsW in a concentration dependent manner. Consistent with the GTase data this inhibitory effect was relieved by the presence of PBP3 (Fig. 3D). The same trend was observed in PG produced by PBP1b in the presence of LpoB, although in this experiment the presence of PBP3 did not fully restore peptide cross-linking to the uninhibited levels.

### Interaction of FtsW with lipid II and its modulation by PBP3

**Figure 4:**
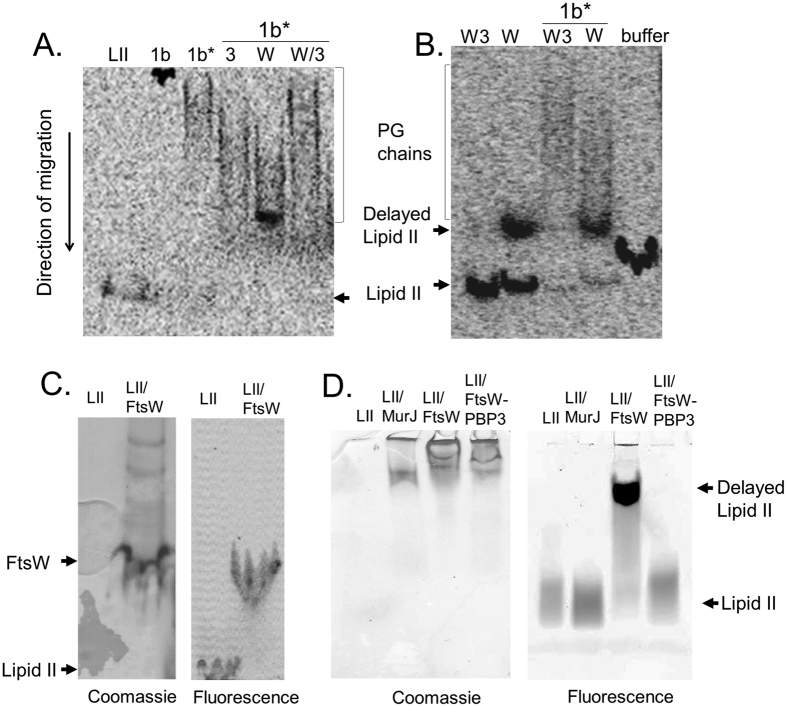
Effect of FtsW, PBP3 and the FtsW-PBP3 complex on the polymerization of lipid II by PBP1b and the mobility of lipid II (LII) analyzed by (SDS- or native) PAGE. (**A** and **B**) radioactive lipid II revealed by storage phosphor imaging using Typhoon trio+ (GE Health Care). (**A**) SDS-PAGE analysis of PBP1b (1b) or PBP1bTP* mutant (1b*) (both at 100 nM) reaction products in the presence of PBP3 (3) (3 μM), FtsW (W) (3 μM), and the FtsW-PBP3 (W3) complex (3 μM). (**B**) effect of FtsW (3 μM) and the FtsW-PBP3 complex (3 μM) in the absence of PBP1b on the mobility of lipid II; buffer with excess detergent used as control. (**C**) Co-migration of FtsW and NBD-lipid II in SDS-PAGE. (**D**) analysis of NBD-lipid II (10 μM) migration by native-PAGE in the absence and the presence of FtsW, the FtsW-PBP3 complex or MurJ (all used at 5 μM). (**C** and **D**) fluorescent lipid II was first revealed by fluorescence imaging using Typhoon trio+, then the gels were stained with Coomassie blue.

A second polymerase assay based on SDS-PAGE and radioactive lipid II was used to further analyze the effect of FtsW and PBP3 on lipid II polymerization by PBP1b. This assay allows the visualization of unused lipid II and the evaluation of the polymer glycan chain length when the TPase active site is inactivated (by the S510A mutation in PBP1bTP* or by penicillin). When both the GTase and TPase are active, the PG does not penetrate the gel, which indicates that it has been cross-linked (Fig. 4A). The PBP1bTP* activity was partially inhibited by addition of FtsW; the glycan chains formed were shorter when compared to the products of PBP1bTP* alone (Fig. 4A). Addition of PBP3 also resulted in a shortening of glycan chains but to a lesser extent than with FtsW (Fig. 4A). Moreover, we observed that FtsW modifies the mobility of lipid II in the gel while the FtsW-PBP3 complex had no effect on the activity of PBP1b or the mobility of lipid II (Fig. 4A and B). Similarly, the addition of FtsW to lipid II in the absence of PBP1b reduced the electrophoretic motility of lipid II in the gel, compared to its migration near the gel front in the sample with the FtsW-PBP3 complex (Fig. 4B). Coomassie staining of the gel shows the co-migration of FtsW and the fluorescent lipid II (Fig. 4C) suggesting a direct interaction between lipid II and FtsW. To further confirm this interaction, we used native PAGE. Interestingly, we found that the mobility of lipid II pre-incubated with FtsW was also affected under native conditions, and that the FtsW-PBP3 complex or MurJ had no effect on the mobility of lipid II (Fig. 4D) showing that the interaction between FtsW and lipid II is specific. These results are consistent with the observation that overproduction of FtsW arrests cell growth^41^. We also observed that high induction of FtsW arrests cell growth at an OD of ~1.5 but when FtsW-PBP3 was overexpressed, cells continue to grow (Fig. S2).

All together our results provide evidence for a direct interaction between lipid II and FtsW and suggest that this interaction is responsible for the inhibition of PBP1b-catalyzed lipid II polymerization by FtsW. Moreover, the results also indicate that the interaction of FtsW with PBP3 modulates the interaction of the transporter candidate with its lipid II substrate.

**Figure 5:**
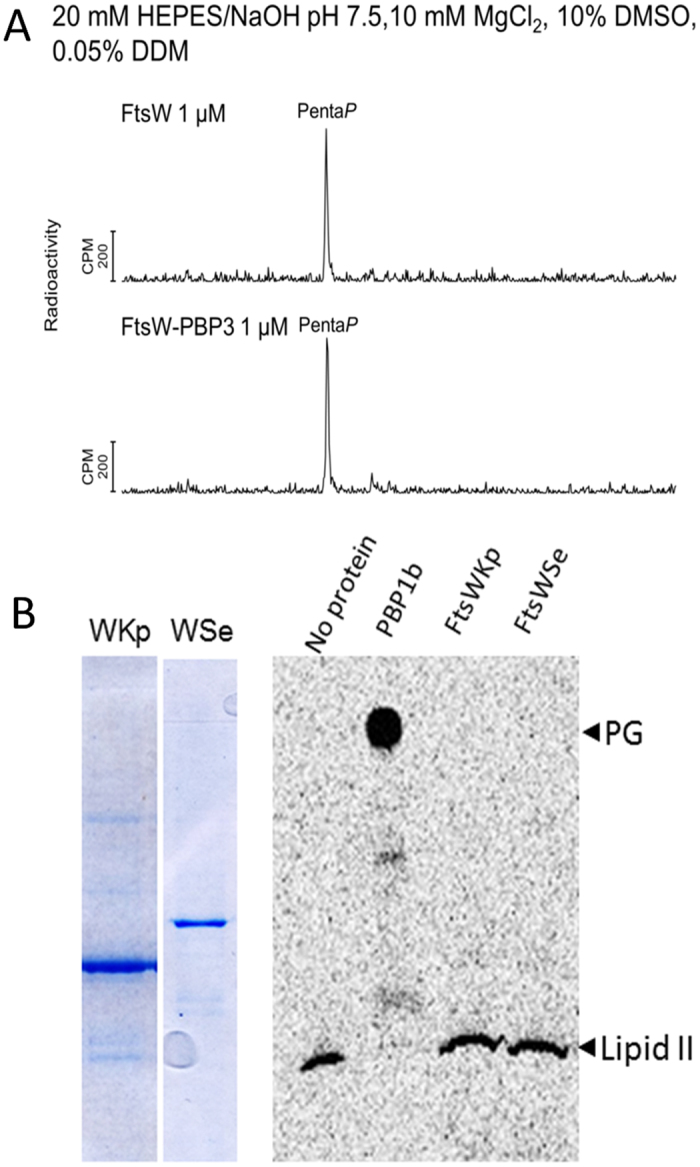
Analysis of the reactivity of FtsW with lipid II. (**A**) HPLC chromatograms of the reaction products of radioactive lipid II incubated with *E. coli* FtsW or FtsW-PBP3. (**B**) right, TLC analysis of reaction products from radioactive lipid II incubated with FtsWKp or FtsWSe. Lipid II without protein and peptidoglycan (PG) synthesis by PBP1b is shown as control. Left, SDS-PAGE showing the purified *K. pneumoniae* FtsW (WKp) and *S. enterica* FtsW (WSe).

While we have observed an interaction of *E. coli* FtsW with lipid II, this protein and two orthologues (*K. pneumoniae* FtsW or *Salmonella enterica* FtsW) were unable to catalyze the GTase reaction *in vitro* (Fig. 5). No polymerase activity was observed with FtsW-PBP3 complex either (Fig. 5A and Fig. S1). This contrasts the recent findings of a weak GT activity of a partially purified RodA protein from *B. subtilis*^22^ and the proposed *in vivo* GTase activity of *E. coli* RodA^42^. Our data suggest that in *E. coli* the GTase activity might be provided by the bi-functional class A PBPs and not by the two SEDS proteins, FtsW and RodA.

### MurJ does not affect lipid II polymerization by PBP1b

The identity of the lipid II flippase is currently a matter of debate^5^. In *E. coli*, the SEDS proteins (FtsW and RodA) and MurJ were proposed as lipid II flippases^3^,^4^. The previous studies focused on the transport function of FtsW and MurJ using different *in vitro* and *in vivo* experiments, respectively. However, the different approaches did not produce consistent results to end the controversies about the identity of the lipid II transporter, which remains an open question. In this study, we have investigated the effect of MurJ on lipid II polymerization by PBP1b as described above for FtsW. We assumed that if MurJ is a lipid II transporter, it should interact with its substrate and thus could affect the activity of PBP1b as was observed with FtsW. When MurJ was added to the fluorescence assay containing PBP1b and dansyl lipid II, we found that, in contrast to the three different FtsW orthologues tested, MurJ has no effect on the polymerization of lipid II by PBP1b (Fig. 3E). Moreover, when MurJ was incubated with lipid II it did not exhibit any effect on the lipid II mobility in a native gel (Fig. 4D). These results suggest that MurJ may not interact directly with lipid II and may require unknown factors should it be a lipid II flippase. As FtsW and MurJ are both integral membrane proteins and were solubilized with the same detergent, our data highlight the specificity of the effect of FtsW on lipid II polymerization by PBP1b.

## Discussion

### New insights from FtsW-PBP3/PBP1b interactions suggest a functional coordination

In this work we have investigated the activities and interactions of core peptidoglycan synthesis proteins of the *E. coli* divisome. Our results show that PBP1b, PBP3 and FtsW form a ternary complex *in vitro*. Such a complex contains the necessary enzymatic activities to build the septum peptidoglycan, namely the GTase and TPase activities for glycan chain elongation and peptide cross-linking, respectively, provided by the bifunctional PBP1b and the division specific TPase activity of PBP3, in addition to the potential flippase activity of FtsW. This reinforces the idea that the three proteins work together during division in coordination with other divisome proteins. Our data is consistent with previous protein-protein interaction and localization data^2^,^8^ and support the hypothesis that class A and class B PBPs (here PBP1b and PBP3) participate in multi-enzyme complexes^43^,^44^. *E. coli* and other bacteria, require at least one class A PBP (PBP1a or PBP1b) for viability^45^,^46^. PBP1b can be replaced by PBP1a and therefore the latter is likely to be also recruited at the division site under specific control of the divisome.

We infer that our method of co-expression/-purification would detect only strong and stable interactions between proteins. Comparing the data from different overexpression pairs provided new insights into the stability of the interactions observed. The FtsW-PBP3 and FtsW-PBP1b complexes were readily isolated suggesting that they are maintained by considerably stronger interactions than those between PBP1b and PBP3. Consequently, PBP1b is likely recruited to the division site by the FtsW-PBP3 complex by multiple and simultaneous interactions, consistent with its PBP3-dependent mid-cell localization^15^. The loop between TM9 and 10 of FtsW is necessary for interaction with PBP3^18^ and may also be involved in the interaction with PBP1b^12^. The largest periplasmic loop between TM7 and 8 (~66 residues) of FtsW is crucial for the function of the protein^18^. We showed here that the modification of this loop decreased the binding of FtsW_HA_ to PBP3 to a point where the complex could no longer be detected, but the formation of the FtsW_HA_-PBP1b complex was not affected. These observations indicate that the loop between TM7 and 8 contributes to the interaction of FtsW with PBP3 but does not seem to play a role in the interaction with PBP1b. This is consistent with interaction studies between FtsW and PBP3 from *Mycobacterium tuberculosis* showing that the last two extracellular loops between TM 7/8 and TM 9/10 of FtsW also play important role in the binding of PBP3^47^.

### PBP3 modulates the interaction between FtsW and lipid II

The finding of the inhibitory effect of FtsW on the polymerization of lipid II by *E. coli* PBP1b, the suppression of the inhibitory effect of FtsW by PBP3, together with the interaction of FtsW with lipid II, strongly suggest a regulation mechanism of FtsW involving PBP3. Based on these results, we propose: (i) that FtsW alone interacts with high affinity with lipid II and prevents its polymerization by PBP1b, (ii) that the interaction of PBP3 with FtsW modulates the interaction between FtsW and lipid II so that the latter becomes accessible to PBP1b. The interaction of PBP3 with FtsW may induce a conformational change in FtsW that facilitates the release of lipid II and/or its transfer to PBP1b. FtsW and PBP3 are expressed at similar levels in cells and the two proteins form a complex prior to their localization at the division site, hence, only the active FtsW-PBP3 complex might be present *in vivo*. This is consistent with the observation that overproduction of FtsW inhibits cell growth^41^, in contrast, the overexpression of FtsW-PBP3 had no effect on the cell growth. The effect of overexpressed FtsW on cell growth may be the consequence of the sequestration of lipid II by the overexpressed protein. Thus, sequestration of lipid II when FtsW is overexpressed and its recovered availability when both FtsW and PBP3 are overexpressed seem to also happen *in vivo*.

In our study we have observed an interaction of *E. coli* FtsW with lipid II but this protein, and two analogs (FtsWKp and FtsWSe), were devoid of any GT activity. This contrasts the observed activity of RodA from *B. subtilis*^22^. These marked differences indicate that in *E. coli*, where at least one class A PBP is essential for viability, the GTase activity might be provided by the bi-functional class A PBPs and not by the two SEDS proteins, FtsW and RodA. Moreover, the activity data, together with the interaction detected between PBP1b and several divisome proteins, argue against the recent suggestion^42^ that the class A PBPs work ‘semi-autonomously’ outside the divisome/elongasome complexes and that the GT activity is provided by the SEDS proteins. In our view, other SEDS proteins need to be purified and systematically tested for GTase activity, lipid II binding and flippase activity to clarify these contradicting results and help better understand the function of these proteins in PG synthesis and cell morphogenesis. More effort should be put into the determination of the structure of SEDS protein, which could provide an insight on their function.

Based on our results and published data we propose a model where FtsW is a flippase interacting with its substrate, lipid II and that this interaction is regulated by PBP3 (see below).

### Model of FtsW function and its regulation by PBP3

**Figure 6:**
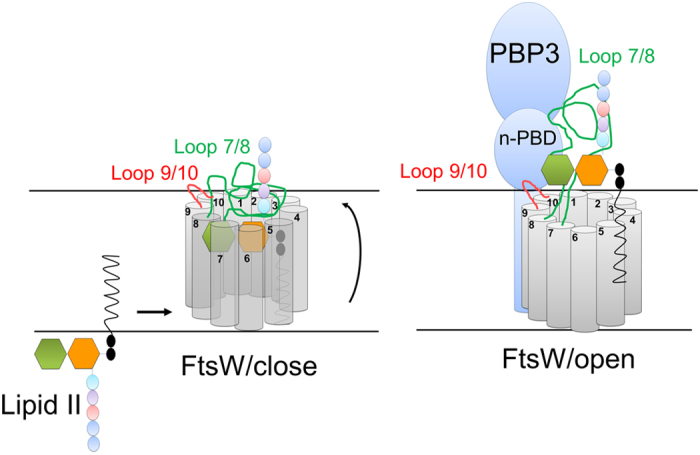
Model of modulation of the FtsW function by PBP3. Left, in the absence of PBP3, lipid II is loaded into the pore of FtsW and translocated but cannot be released. Right, Interaction of PBP3 with FtsW induces conformational changes in the loop between TM 7 and 8, opening the pore gate and allowing the release of lipid II. The loop between TMs 9 and 10 is required for the localisation of PBP3 at the septum.

The potential transport function of FtsW was proposed to be mediated by a pore-like structure^17^. The large loop between TM7 and 8 could thus play a regulatory role by an open/close mechanism of the pore’s gate from the periplasmic side. As this loop interacts with PBP3, possibly via the N-terminal non-penicillin binding domain (n-PBD), this may induce a conformational change of the TM7/8 loop and opening of the pore, allowing the release of lipid II and/or its direct transfer to PBP1b. This mechanism would explain the observed effect of PBP3 on the interaction of FtsW with lipid II (Fig. 6). Alternatively, PBP3 binding could induce a structural rearrangement within the TM domain of FtsW leading to the lipid II release.

A similar mechanism is found in functionally unrelated transferases that use a polyprenyl–sugar carrier as substrate^48^,^49^. The archaeal AglB and the eubactertial PglB, two oligosaccharyltransferase (OST) belonging to family STT3 (stauroporine and temperature sensitive 3), catalyze protein N-glycosylation using polyprenyl-phospho-sugar as a donor. Modeling showed that their folds were related to that of FtsW^34^. They contain a characteristic long and dynamic loop between TM9 and 10 that oscillates between structured and unstructured states, facilitating the release of the glycosylated product from the active site^48^. ArnT is an OST with a fold similar to that of PglB and AglB but its function is different. ArnT is involved in the attachment of the undecaprenyl-linked L-Ara4N to lipid A. ArnT also exhibits a flexible periplasmic loop between TM7 and 8 that plays an important function by controlling the lipid A cavity by a structural change^49^.

FtsW and MurJ are both proposed to transport lipid II across the membrane that implicates that they should interact with their substrate. In consequence, this interaction should have an effect on the lipid II polymerization by the glycosyltransferase activity of PBP1b. This hypothesis was verified with FtsW but, in contrast, MurJ had no effect on the activity of PBP1b nor did it bind lipid II. This indicates that the two potential flippases work by different mechanisms. The modulation of the interaction between FtsW and lipid II by PBP3 could stimulate the flippase activity of FtsW. The lack of an interaction of MurJ with lipid II suggests that MurJ may have an indirect function in the process or that an unknown factor may be required for binding and probably the flippase activity, which would explain why MurJ alone was not active in the *in vitro* assay^3^. Alternatively, the turnover of lipid II by MurJ is fast and the release of lipid II does not require assistance from another protein.

In conclusion, we showed that FtsW, PBP3 and PBP1b form a ternary complex *in vitro*, FtsW interacts with lipid II and that this interaction is modulated by PBP3. We also showed that the loop between TM 7 and 8 is important for the interaction of FtsW with PBP3 and that this interaction may play role in the regulation of FtsW-lipid II interaction. Based on these results we propose a model where lipid II remains attached to FtsW after its transport across the cytoplasmic membrane and that its release and/or transfer to PBP1b is facilitated by PBP3 via allosteric conformational changes probably involving the loop between TM 7 and 8 of FtsW and/or the TM domain. This regulatory mechanism may ensure that the limited and precious lipid II precursor is not spuriously flipped to a non-productive end.

## Experimental Procedures

### Bacterial strains, plasmids and growth conditions

Bacterial strains and plasmids are described in Table 1. Plasmids constructions are described in the supporting information. Oligonucleotides (Table S1) were purchased from Eurogentec (Angleur, Belgium). Bacteria were grown in Luria-Bertani (LB) medium supplemented with ampicillin (50 μg/ml) (from MP Biomedicals) or chloramphenicol (30 μg/ml) (from Sigma) or Kanamycin (50 μg/ml) (from MP Biomedicals).

### Reagents

Dansyl-lipid II, NBD-lipid II and [^14^C]-lipid II (0.155 μCi/nmol) were prepared as previously described^50^,^51^, Flavomycin was a gift from Aventis (Romainville, France). Fluorescein labelled ampicillin was prepared as previously described^52^.

### Expression and purification of the proteins

*E. coli* PBP1b and PBP1b-S510A were expressed and purified as previously described^50^. LpoB (sol) was purified as described^25^.

*E. coli* FtsW, PBP3 or the PBP3-FtsW complex were expressed in *E. coli* strain C43 (DE3) harboring plasmids pDML2400, pDML2040, pDML2041 or pDML2043. Bacteria were grown at 37 °C, in LB medium supplemented with the appropriate antibiotic to an A_600nm_ of 0.6. Then expression was induced for 4–5 hours by addition of 1 mM isopropyl β-D-1-thiogalactopyranoside (IPTG). Cells were collected by centrifugation at 8000 × *g* for 20 minutes at 15 °C and resuspended in a buffer containing 50 mM Tris–HCl, pH 8.0, 50 mM NaCl, 2 mM MgCl_2_, 1.5 U ml^−1^ benzonase (EMD Millipore) and EDTA-free protease inhibitor Cocktail (Roche). The bacterial cells were lysed by three passages through a cell homogenizer (Emulsiflex C3 Avestin^®^). The cell lysate was spun down at 150,000× g for 1 hour at 4 °C and then the membranes were resuspended and washed in 25 mM Tris–HCl, pH 8.0, 500 mM NaCl, 10% glycerol and complete EDTA-free protease inhibitors. The membranes were collected by centrifugation at 150,000× g for 1 hour at 4 °C and solubilized in 50 mM Hepes pH 7.5, 500 mM NaCl, 10% glycerol, 40 mM *n*-dodecyl-β-D-maltopyranoside (DDM; Inalco^®^) and complete EDTA-free protease inhibitors. The mixture was incubated for 1 hour at room temperature followed by centrifugation at 150,000× g for 1 hour at 4 °C, the supernatant containing the solubilized membrane proteins or complexes were purified by Ni-affinity chromatography.

The samples were loaded onto a HisTrap column (GE HealthCare) conditioned in buffer A (50 mM Hepes, pH 7.5, 300 mM NaCl, 10% glycerol, 50 mM imidazole and 1 mM DDM). The proteins or complexes of proteins were eluted by an imidazole gradient (0.05–1 M). After SDS-PAGE analysis, pure fractions were pooled and desalted on a G25 Sephadex column and concentrated using an Amicon apparatus (EMD Millipore) with a 50 kDa cutoff membrane (for PBP3 and FtsW) or a 100 kDa cutoff (for the complex) and stored at −20 °C. The concentrations of the proteins or complexes were determined with the help of the BCA reagent kit (ThermoFisher Scientific).

The expression and purification procedures of the His-tagged FtsW-PBP1b and the ternary HisPBP3-FtsW-PBP1b complexes were similar to those described above, except that the membranes were solubilized with 40 mM of Lauryl Maltose Neopentyl Glycol (LMNG, Anatrace) instead of DDM and that the purification was carried out in buffer B (50 mM Hepes, pH 7.5, 200 mM NaCl, 10% glycerol and 0.2 mM LMNG).

Before the analysis of the fractions containing PBPs by SDS-PAGE, the samples were labelled with fluorescent ampicillin at 10 μM for 30 minutes at 37 °C.

FtsWs from *Klebsiella pneumoniae* and *Salmonella enterica* were expressed in *E. coli* Lemo21 (DE3) strain (New England Biolabs, Ipswich, MA). Cells were grown at 37 °C in LB medium until the cell density reached an A_600nm_ of 0.4. The temperature was then decreased to 22 °C before induction with 0.8 mM IPTG and 100 μM rhamnose. After growth at 22 °C for 16 h, cells were harvested by centrifugation at 8000 × *g*. Cells were resuspended in cold lysis buffer (50 mM Tris-HCl pH 7.5, 500 mM NaCl, 5% glycerol, 5 mM MgCl_2_ and 2 mM β-mercaptoethanol) and disrupted by a homogeneizer (Emulsiflex C3 Avestin^®^). The cell lysate was centrifuged at 15,000× *g* for 15 min at 4 °C, and the supernatant was then ultracentrifuged at 150,000× g for 1 hour. The membrane fractions were resuspended in buffer C (25 mM Sodium Phosphate pH 7, 500 mM NaCl, 5% (vol/vol) glycerol and 2 mM β-mercaptoethanol) supplemented with 40 mM DDM. After another ultracentrifugation at 150,000 × g for 30 min, the supernatant was collected and purified by affinity chromatography on a HisTrap column (GE Healthcare). Fractions containing the purified protein were pooled, desalted on a Sephadex G25 column equilibrated with buffer C, concentrated using the Amicon apparatus with 50 kDa cutoff membrane (EDM Millipore) and stored at −20 °C.

*E. coli* MurJ was expressed in *E. coli* C43 (DE3) and purified on the Ni-NTA column as described above except that the expression was induced with 0.2 mM ITPG and that the protein purified in 25 mM Tris-HCl pH 7.5, 100 mM NaCl, 5% glycerol and 2 mM β-mercaptoethanol.

### Continuous fluorescence assay

The PBP1b activity assays with dansyl lipid II as substrate were performed as described^36^,^37^ in a medium binding black 96-well microplate (Greiner Bio One). The samples contained 10 μM dansyl-lipid II, 50 mM Hepes pH 7.5, 200 mM NaCl, 10 mM CaCl_2_, 0.02% of decyl-PEG, 20% of dimethylsulfoxide (DMSO), 1000 unit ml^−1^ of penicillin G, when required, and 1 unit of *N*-acetylmuramidase of *Streptococcus globisporus* (Calbiochem). The proteins FtsW, PBP3, the FtsW-PBP3 complex or MurJ, were used at 0.5–5 μM concentrations and LpoB at 200 nM. The reactions were initiated by the addition of 50 nM PBP1b or PBP1b (S510A). The reaction was monitored by following the fluorescence decrease over 20–30 min at 30 °C using an Infinite 200 PRO Microplate reader (Tecan, Männedorf, Switzerland) with excitation at 340 nm and emission at 520 nm.

### Analysis of glycan chains by SDS-PAGE

*In vitro* PG polymerization from radioactive lipid II was performed in 50 mM Hepes pH 7.5, 10 mM CaCl_2_, 200 mM NaCl, 0.02% decyl-PEG, 10% DMSO, 4 μM [^14^C]-lipid II (0.155 μCi. nmol^−1^) and 100 nM PBP1b or PBP1b (S510A). FtsW, PBP3, and the FtsW-PBP3 complex or MurJ were used at 3 μM. The samples were incubated at 37 °C for 1 hour and the reactions were stopped by the addition of Flavomycin (5 mM). The reaction products were analyzed by 9% SDS-PAGE as described^53^. The gels were dried, exposed to a storage phosphor screen (GE Healthcare) and imaged with a Typhoon Trio+ (GE Healthcare).

The interactions of lipid II with FtsW complex was also analyzed using the same procedure with modification: SDS was omitted in the gel and the loading buffer and was only added in the running buffer.

### Analysis of the reactivity of FtsW with lipid II by TLC

4 μM [^14^C]-lipid II was incubated with 2 μM of purified *E. coli, K. penumoniae* or *S. enterica* FtsW proteins in 50 mM Hepes/NaOH pH7.0, 20 mM MgCl_2_, 20 mM CaCl_2_, 20% DMSO and 0.05% DDM for 2 h at 30 °C. PBP1b (100 nM) was incubated in the same condition as control for PG synthesis. The reaction products were separated by thin-layer chromatography (TLC) on silica gel plates (Fluka), using 2-propanol–ammonium hydroxide (25%)–water (6:3:1, vol/vol/vol) as the mobile phase. The TLC plates were exposed to a storage phosphor screen (GE Healthcare) for 16 h, and images were revealed using a Typhoon Trio imager. The images were analyzed using Image Quant TL software (GE Healthcare).

### *In vitro* peptidoglycan synthesis assay with HPLC analysis of product

Performed as described previously^54^ with changes to the buffer conditions. Reactions were performed either in a solution of 10 mM Hepes/NaOH, 3 mM MgCl_2_, 200 mM NaCl, 0.04% DDM (w/v), pH 7.5 or in 20 mM Hepes/NaOH, 10 mM MgCl_2_, 25 mM NaCl, 10% DMSO, 0.05% DDM (w/v), pH 7.5. Lipid II substrate was dried and re- dissolved in 5 μL of 0.1% DDM prior to mixing with the appropriate enzyme/protein mixtures to give 15 μM final concentration. Combinations of 0.5 μM PBP1b with 2 or 5 μM of FtsW or FtsW-PBP3 complex in the presence or absence of 2 μM LpoB (sol). Reaction proceeded for 2 h at 37 °C. Processing of PG product for HPLC analysis was as described^54^.

### Analysis of lipid II-proteins interaction by Native-PAGE

Native-PAGE was performed essentially as described^55^ using a 4–15% gel gradient in glycine buffer pH 10.6. The samples: NBD-lipid II (10 μM), or lipid II-protein (10 μM/5 μM) mixtures containing FtsW, the FtsW-PBP3 complex or MurJ were supplemented with 5% sucrose before electrophoresis. The gels were imaged with a Typhoon Trio+ (GE Healthcare) followed by Coomassie blue staining.

### Western blotting

After SDS-PAGE, the proteins were electro-transferred to a PVDF membrane and probed by incubation with monoclonal anti-HA antibodies (Abcam). Immuno detection was done using peroxidase conjugated goat anti-mouse IgG secondary antibodies (Millipore). The proteins were visualized by enhanced chemiluminescence (ECL kit, GE Healthcare).

## Additional Information

**How to cite this article:** Leclercq, S. *et al*. Interplay between Penicillin-binding proteins and SEDS proteins promotes bacterial cell wall synthesis. *Sci. Rep.* **7**, 43306; doi: 10.1038/srep43306 (2017).

**Publisher's note:** Springer Nature remains neutral with regard to jurisdictional claims in published maps and institutional affiliations.

## Supplementary Material

Supporting Information

## Figures and Tables

**Figure 1:**
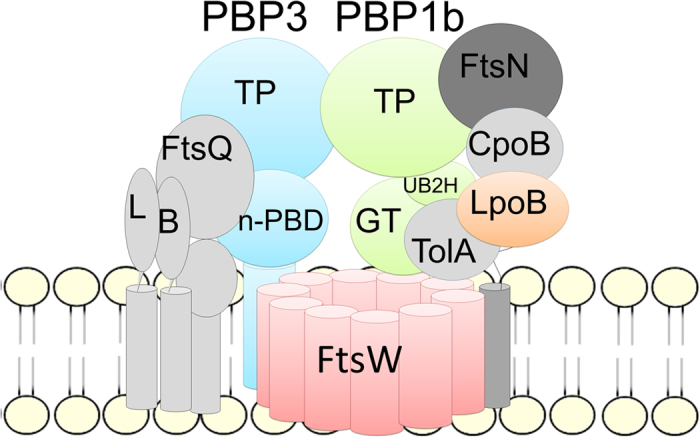
Schematic representation of the septal peptidoglycan synthesis core of the divisome. The proteins studied in this paper are shown in color, the others are in grey. TP, transpeptidase domain; GT, glycosyltransferase domain; L, FtsL; B, FtsB; n-PBD, non-penicillin binding domain.

**Table 1:** Strains and plasmids constructs.

Strain/plasmids	Relevant genetic marker (s) or features	Sources/reference
***E. coli*** **Strains**		
Mach-I	*ΔrecA1398 endA1 tonA Φ80ΔlacM15 ΔlacX74 hsdR (r*_*K*_^−^_*K*_^+^)	Invitrogen
C43 (DE3)	*F*^*−*^ *ompT gal dcm hsdS*_*B*_ (*r*_*B*_^−^ *m*_*B*_^−^) (*DE3*)	56
Lemo21 (DE3)	*fhuA2 [lon] ompT gal (λ DE3*) [*dcm*] *∆hsdS*/*pLemo* (Cam^R^)	New England Biolabs
**Plasmids**		
pDML924	pET28a, His-*ponB*	50
pDML924 (S510A)	pET28a, His-*ponB* (S510A)	This work
pDML2032	pET22b, *ftsN*-His	This work
pDML2422	pMCL210*-fts*W-293HA	18
pDML2400	pET28a, His-*ftsW*	18
pDML2040	pETDuet, His-*ftsI, ftsW*	This work
pDML2041	pETDuet, His-*ftsW, ftsI*	This work
pDML2043	pETDuet, His-*ftsI, ftsW* (293HA)	This work
pDML2045	pACYCDuet, *ponB, ftsN-S. tag*	This work
pCIP1000	pETDuet, His-*ftsW, ponB*	This work
pCIP1083	pETDuet, His-*ftsW*_HA_, *ponB*	This work
pET28MHL*-*WSe	pET28MHL, His*-ftsWSe*	This work
pET28MHL*-*W*Kp*	pET28MHL, His*-ftsWKp*	This work
pCIP1051	pETDuet, His*-murJ*	This work

## References

[b1] Vollmer W., Blanot D. & de Pedro M. A. Peptidoglycan structure and architecture. FEMS Microbiol Rev 32, 149–167 (2008). 18194336 10.1111/j.1574-6976.2007.00094.x

[b2] Egan A. J. F. & Vollmer W. The physiology of bacterial cell division. Ann N Y Acad Sci 1277, 8–28 (2013). 23215820 10.1111/j.1749-6632.2012.06818.x

[b3] Mohammadi T. . Identification of FtsW as a transporter of lipid-linked cell wall precursors across the membrane. EMBO J 30, 1425–32 (2011). 21386816 10.1038/emboj.2011.61 PMC3102273

[b4] Sham L.-T. . Bacterial cell wall. MurJ is the flippase of lipid-linked precursors for peptidoglycan biogenesis. Science 345, 220–2 (2014). 25013077 10.1126/science.1254522 PMC4163187

[b5] Ruiz N. Lipid Flippases for Bacterial Peptidoglycan Biosynthesis. Lipid Insights 8, 21–31 (2015). 26792999 10.4137/LPI.S31783 PMC4714577

[b6] Sauvage E. & Terrak M. Glycosyltransferases and Transpeptidases/Penicillin-Binding Proteins: Valuable Targets for New Antibacterials. Antibitics 5, 12 (2016). 10.3390/antibiotics5010012 PMC4810414 27025527

[b7] Sauvage E., Kerff F. F., Terrak M., Ayala J. A. & Charlier P. The penicillin-binding proteins: structure and role in peptidoglycan biosynthesis. FEMS Microbiol Rev 32, 234–258 (2008). 18266856 10.1111/j.1574-6976.2008.00105.x

[b8] Gray A. N. . Coordination of peptidoglycan synthesis and outer membrane constriction during Escherichia coli cell division. Elife 4 (2015). 10.7554/eLife.07118 PMC4458516 25951518

[b9] Gerding M. A., Ogata Y., Pecora N. D., Niki H. & de Boer P. A. J. The trans-envelope Tol-Pal complex is part of the cell division machinery and required for proper outer-membrane invagination during cell constriction in E. coli. Mol Microbiol 63, 1008–25 (2007). 17233825 10.1111/j.1365-2958.2006.05571.x PMC4428343

[b10] Aarsman M. E. . Maturation of the Escherichia coli divisome occurs in two steps. Mol Microbiol 55, 1631–1645 (2005). 15752189 10.1111/j.1365-2958.2005.04502.x

[b11] Buddelmeijer N. & Beckwith J. A complex of the Escherichia coli cell division proteins FtsL, FtsB and FtsQ forms independently of its localization to the septal region. Mol Microbiol 52, 1315–1327 (2004). 15165235 10.1111/j.1365-2958.2004.04044.x

[b12] Fraipont C. . The integral membrane FtsW protein and peptidoglycan synthase PBP3 form a subcomplex in Escherichia coli. Microbiology 157, 251–9 (2011). 20847002 10.1099/mic.0.040071-0

[b13] Goehring N. W., Gonzalez M. D. & Beckwith J. Premature targeting of cell division proteins to midcell reveals hierarchies of protein interactions involved in divisome assembly. Mol Microbiol 61, 33–45 (2006). 16824093 10.1111/j.1365-2958.2006.05206.x

[b14] Mercer K. L. N. & Weiss D. S. The Escherichia coli cell division protein FtsW is required to recruit its cognate transpeptidase, FtsI (PBP3), to the division site. J Bacteriol 184, 904–12 (2002). 11807049 10.1128/jb.184.4.904-912.2002 PMC134820

[b15] Bertsche U. . Interaction between two murein (peptidoglycan) synthases, PBP3 and PBP1B, in Escherichia coli. Mol Microbiol 61, 675–690 (2006). 16803586 10.1111/j.1365-2958.2006.05280.x

[b16] Müller P. . The Essential Cell Division Protein FtsN Interacts with the Murein (Peptidoglycan) Synthase PBP1B in Escherichia coli. J Biol Chem 282, 36394–36402 (2007). 17938168 10.1074/jbc.M706390200

[b17] Mohammadi T. . Specificity of the transport of lipid II by FtsW in Escherichia coli. J Biol Chem 289, 14707–18 (2014). 24711460 10.1074/jbc.M114.557371 PMC4031526

[b18] Pastoret S. . Functional analysis of the cell division protein FtsW of Escherichia coli. J Bacteriol 186, 8370–8379 (2004). 15576787 10.1128/JB.186.24.8370-8379.2004 PMC532424

[b19] Hvorup R. N. . The multidrug/oligosaccharidyl-lipid/polysaccharide (MOP) exporter superfamily. Eur J Biochem 270, 799–813 (2003). 12603313 10.1046/j.1432-1033.2003.03418.x

[b20] Elhenawy W. . The O-Antigen Flippase Wzk Can Substitute for MurJ in Peptidoglycan Synthesis in Helicobacter pylori and Escherichia coli. PLoS One 11, e0161587 (2016). 27537185 10.1371/journal.pone.0161587 PMC4990322

[b21] Meeske A. J. . MurJ and a novel lipid II flippase are required for cell wall biogenesis in Bacillus subtilis. Proc Natl Acad Sci USA 112, 6437–42 (2015). 25918422 10.1073/pnas.1504967112 PMC4443310

[b22] Meeske A. J. . SEDS proteins are a widespread family of bacterial cell wall polymerases. Nature 537, 634–638 (2016). 27525505 10.1038/nature19331 PMC5161649

[b23] Paradis-Bleau C. . Lipoprotein cofactors located in the outer membrane activate bacterial cell wall polymerases. Cell 143, 1110–20 (2010). 21183074 10.1016/j.cell.2010.11.037 PMC3085243

[b24] Typas A. . Regulation of peptidoglycan synthesis by outer-membrane proteins. Cell 143, 1097–109 (2010). 21183073 10.1016/j.cell.2010.11.038 PMC3060616

[b25] Egan A. J. F. . Outer-membrane lipoprotein LpoB spans the periplasm to stimulate the peptidoglycan synthase PBP1B. Proc Natl Acad Sci USA 111, 8197–202 (2014). 24821816 10.1073/pnas.1400376111 PMC4050580

[b26] Liu B., Persons L., Lee L. & de Boer P. A. J. Roles for both FtsA and the FtsBLQ subcomplex in FtsN-stimulated cell constriction in Escherichia coli. Mol Microbiol 95, 945–70 (2015). 25496160 10.1111/mmi.12906 PMC4428282

[b27] Tsang M.-J. & Bernhardt T. G. A role for the FtsQLB complex in cytokinetic ring activation revealed by an ftsL allele that accelerates division. Mol Microbiol 95, 925–44 (2015). 25496050 10.1111/mmi.12905 PMC4414402

[b28] Trip E. N. & Scheffers D.-J. A 1 MDa protein complex containing critical components of the Escherichia coli divisome. Sci Rep 5, 18190 (2015). 26643979 10.1038/srep18190 PMC4672292

[b29] Noirclerc-Savoye M. . Reconstitution of membrane protein complexes involved in pneumococcal septal cell wall assembly. PLoS One 8, e75522 (2013). 24147156 10.1371/journal.pone.0075522 PMC3798694

[b30] Di Lallo G., Fagioli M., Barionovi D., Ghelardini P. & Paolozzi L. Use of a two-hybrid assay to study the assembly of a complex multicomponent protein machinery: bacterial septosome differentiation. Microbiology 149, 3353–9 (2003). 14663069 10.1099/mic.0.26580-0

[b31] Karimova G., Dautin N. & Ladant D. Interaction network among Escherichia coli membrane proteins involved in cell division as revealed by bacterial two-hybrid analysis. J Bacteriol 187, 2233–43 (2005). 15774864 10.1128/JB.187.7.2233-2243.2005 PMC1065216

[b32] Piette A. . Structural determinants required to target penicillin-binding protein 3 to the septum of Escherichia coli. J Bacteriol 186, 6110–7 (2004). 15342580 10.1128/JB.186.18.6110-6117.2004 PMC515155

[b33] Wissel M. C., Wendt J. L., Mitchell C. J. & Weiss D. S. The transmembrane helix of the Escherichia coli division protein FtsI localizes to the septal ring. J Bacteriol 187, 320–8 (2005). 15601716 10.1128/JB.187.1.320-328.2005 PMC538840

[b34] Ovchinnikov S. . Large scale determination of previously unsolved protein structures using evolutionary information. Elife 4, e09248 (2015). 26335199 10.7554/eLife.09248 PMC4602095

[b35] Khattar M. M. . Two polypeptide products of the Escherichia coli cell division gene ftsW and a possible role for FtsW in FtsZ function. J Bacteriol 179, 784–93 (1997). 9006034 10.1128/jb.179.3.784-793.1997 PMC178761

[b36] Schwartz B., Markwalder J. A., Seitz S. P., Wang Y. & Stein R. L. A kinetic characterization of the glycosyltransferase activity of Eschericia coli PBP1b and development of a continuous fluorescence assay. Biochemistry 41, 12552–12561 (2002). 12369847 10.1021/bi026205x

[b37] Egan A. J. F. & Vollmer W. Continuous Fluorescence Assay for Peptidoglycan Glycosyltransferases. Methods Mol Biol 1440, 171–84 (2016). 27311672 10.1007/978-1-4939-3676-2_13

[b38] Bertsche U., Breukink E., Kast T. & Vollmer W. *In vitro* murein peptidoglycan synthesis by dimers of the bifunctional transglycosylase-transpeptidase PBP1B from Escherichia coli. J Biol Chem 280, 38096–38101 (2005). 16154998 10.1074/jbc.M508646200

[b39] Born P., Breukink E. & Vollmer W. *In vitro* synthesis of cross-linked murein and its attachment to sacculi by PBP1A from Escherichia coli. J Biol Chem 281, 26985–26993 (2006). 16840781 10.1074/jbc.M604083200

[b40] Egan A. J. F., Biboy J., van’t Veer I., Breukink E. & Vollmer W. Activities and regulation of peptidoglycan synthases. Philos Trans R Soc B Biol Sci 370, 20150031 (2015). 10.1098/rstb.2015.0031 PMC4632607 26370943

[b41] Khattar M. M., Begg K. J. & Donachie W. D. Identification of FtsW and characterization of a new ftsW division mutant of Escherichia coli. J Bacteriol 176, 7140–7 (1994). 7961485 10.1128/jb.176.23.7140-7147.1994 PMC197100

[b42] Cho H. . Bacterial cell wall biogenesis is mediated by SEDS and PBP polymerase families functioning semi-autonomously. Nat Microbiol 1, 16172 (2016). 10.1038/nmicrobiol.2016.172 PMC5030067 27643381

[b43] Höltje J. V. Growth of the stress-bearing and shape-maintaining murein sacculus of Escherichia coli. Microbiol Mol Biol Rev 62, 181–203 (1998). 9529891 10.1128/mmbr.62.1.181-203.1998 PMC98910

[b44] Banzhaf M. . Cooperativity of peptidoglycan synthases active in bacterial cell elongation. Mol Microbiol 85, 179–194 (2012). 22606933 10.1111/j.1365-2958.2012.08103.x

[b45] Yousif S. Y., Broome-Smith J. K. & Spratt B. G. Lysis of Escherichia coli by beta-lactam antibiotics: deletion analysis of the role of penicillin-binding proteins 1A and 1B. J Gen Microbiol 131, 2839–45 (1985). 3906031 10.1099/00221287-131-10-2839

[b46] Reed P., Veiga H., Jorge A. M., Terrak M. & Pinho M. G. Monofunctional transglycosylases are not essential for Staphylococcus aureus cell wall synthesis. J Bacteriol 193, 2549–2556 (2011). 21441517 10.1128/JB.01474-10 PMC3133172

[b47] Datta P. . Interaction between FtsW and penicillin-binding protein 3 (PBP3) directs PBP3 to mid-cell, controls cell septation and mediates the formation of a trimeric complex involving FtsZ, FtsW and PBP3 in mycobacteria. Mol Microbiol 62, 1655–73 (2006). 17427288 10.1111/j.1365-2958.2006.05491.x

[b48] Matsumoto S. . Crystal structures of an archaeal oligosaccharyltransferase provide insights into the catalytic cycle of N-linked protein glycosylation. Proc Natl Acad Sci USA 110, 17868–73 (2013). 24127570 10.1073/pnas.1309777110 PMC3816453

[b49] Petrou V. I. . Structures of aminoarabinose transferase ArnT suggest a molecular basis for lipid A glycosylation. Science (80-) 351, 608–612 (2016). 10.1126/science.aad1172 PMC4963604 26912703

[b50] Terrak M. . The catalytic, glycosyl transferase and acyl transferase modules of the cell wall peptidoglycan-polymerizing penicillin-binding protein 1b of Escherichia coli. Mol Microbiol 34, 350–364 (1999). 10564478 10.1046/j.1365-2958.1999.01612.x

[b51] Breukink E. . Lipid II is an intrinsic component of the pore induced by nisin in bacterial membranes. J Biol Chem 278, 19898–19903 (2003). 12663672 10.1074/jbc.M301463200

[b52] Lakaye B. . Synthesis, purification and kinetic properties of fluorescein-labelled penicillins. Biochem J 300 Pt 1, 141–145 (1994). 8198525 10.1042/bj3000141 PMC1138135

[b53] Barrett D. . Analysis of glycan polymers produced by peptidoglycan glycosyltransferases. J Biol Chem 282, 31964–31971 (2007). 17704540 10.1074/jbc.M705440200 PMC4048933

[b54] Biboy J., Bui N. K. & Vollmer W. *In vitro* peptidoglycan synthesis assay with lipid II substrate. Methods Mol Biol 966, 273–88 (2013). 23299741 10.1007/978-1-62703-245-2_17

[b55] Gallagher S. R. One-dimensional electrophoresis using nondenaturing conditions. Curr Protoc Mol Biol Chapter 10, Unit 10.2B (2001). 10.1002/0471142727.mb1002bs47 18265064

[b56] Miroux B. & Walker J. E. Over-production of proteins in Escherichia coli: mutant hosts that allow synthesis of some membrane proteins and globular proteins at high levels. J Mol Biol 260, 289–298 (1996). 8757792 10.1006/jmbi.1996.0399

