# Supplementary material for: Interplay between Penicillin-binding proteins and SEDS proteins promotes bacterial cell wall synthesis
Source: Sci Rep. 2017 Feb 24;7:43306. doi: 10.1038/srep43306 (PMC5324115; doi:10.1038/srep43306)
Supplement: Supporting Information [file srep43306-s1.pdf]

Supplementary information

Interplay between Penicillin-binding proteins and SEDS proteins promotes bacterial cell wall synthesis

Sophie Leclercq[1], Adeline Derouaux[1], Samir Olatunji[1], Claudine Fraipont[1], Alexander J. F. Egan[2], Waldemar Vollmer[2], Eefjan Breukink[3] and Mohammed Terrak[1]

[1] Centre d'Ingénierie des Protéines, University of Liège, B6a, Quartier Agora, allée du six Août 11, 4000 Liège 1, Belgium

[2] Institute for Cell and Molecular Biosciences, The Centre for Bacterial Cell Biology, Newcastle University, Richardson Road, Newcastle upon Tyne, NE2 4AX, UK

[3] Membrane Biochemistry and Biophysics, Department of Chemistry, Faculty of Science, Utrecht University, Padualaan 8, 3584 CH Utrecht, The Netherlands

To whom correspondence should be addressed:
Mohammed Terrak, Centre d'Ingénierie des Protéines, University of Liège, B6a, Quartier Agora, Allée du six Août 11, 4000 Liège 1, Belgium, Tel.: +33-4366-3332; E-mail: mterrak@ulg.ac.be

Supplementary methods

Plasmid constructions

pDML924 (S510A). Mutation of the active serine S510A in the transpeptidase domain of PBP1b was generated using the QuikChange Site-Directed Mutagenesis method (Agilent Technologies), oligonucleotides S510A1 and S510A2 (Table S1) and plasmid pDML924 [1] as template.

pDML2040. The *ftsI* gene was amplified by PCR from pDML2494 [2] using the IW1 and IW2 primers (Table S1) and inserted in the MCS1 of the pETDuet-1 vector (Novagen-EMD Millipore) between the BamHI and HindIII sites. The *ftsw* gene was amplified by PCR from pDML2400 [3] using the IW3 and IW4 primers (Table S1) and inserted in the MCS2 of the pETDuet-1 vector between the BglII and XhoI sites. This construct allows the production of His-tagged PBP3 and untagged FtsW.

pDML2041. The *ftsW* gene was amplified by PCR from pDML2400 using the WI1 and WI2 primers (Table S1) and inserted in the MCS1 of the pETDuet-1 vector between the BamHI and HindIII sites. The *ftsI* gene was amplified by PCR from pDML2494 [2] using the WI3 and WI4 primers (Table S1) and inserted in the MCS2 of the pETDuet-1 vector between the BglII and XhoI sites. This construct allows the production of His-tag FtsW and untagged PBP3.

pDML2043. Plasmid pDML2422 [3] contains the *fts*W gene with a fragment encoding the antigenic HA peptide (YPYDVPDYA), derived from Human influenza hemagglutinin (HA), inserted between codons for E293 and A294 of the large periplasmic loop. The fragment of the $ftsW_{HA}$ gene between SnaBI-SalI sites and containing the sequence coding for HA peptide was recovered by digestion from the plasmid pDML2422 and inserted at the same sites of plasmid

pDML2040 yielding pDML2043. This construct allows the production of His-tagged PBP3 and untagged $FtsW_{HA}$.

pDML2045. The *ponB* gene was amplified by PCR from pHK2414 [1] using the SN1b1 and SN1b2 primers (Table S1) and inserted in the MCS1 of the pACYCDuet-1 (Novagen-EMD Millipore) between the NcoI and BamHI sites. The *ftsN* gene was obtained by digestion of pDML2032 by NdeI and XhoI and inserted at the same sites in the MCS2 of pACYCDuet-1. This construct allows the production of untagged PBP1b and FtsN-S·tag.

pDML2032. The *fts*N gene was amplified by PCR from pDML2000 [4] using the Nhis1 and Nhis2 primers (Table S1), digested by NdeI and XhoI and introduced in pET22b between the same sites. This construct allows the production of FtsN-His-tag.

pCIP1000. The *ponB* gene was amplified by PCR using the W1b1 and W1b2 primers (Table S1). The resulting fragment was digested by NdeI/XhoI and used to replace the *fts*I gene of plasmid pDML2041 digested with the same enzymes. This construct allows the production of His-tagged FtsW and untagged PBP1b.

pCIP1083. The *ponB* gene was amplified from plasmid pCIP1000 using the W1b1 and W1b2 primers (Table S1) digested by NdeI/XhoI and cloned into the same sites of the MCS2 of an empty pETDuet-1 vector to generate pCIP1061 (pETDuet-*pon*B). Then, the sequence coding for $FtsW_{HA}$ was amplified by PCR from pDML2043 using the WHA1 and WHA2 primers. The PCR product was digested by BamHI/EcoRI and inserted into MCS1 of pCIP1061 between the corresponding sites to give rise to pCIP1083. This construct allows the production of His-tagged $FtsW_{HA}$ and untagged PBP1b.

pET28MHL-W$_{Kp}$ *and* pET28MHL-W$_{Se}$. The genes coding for FtsW *of Klebsiella pneumoniae and Salmonella enterica* were amplified by PCR using the genomic DNAs of the respective strains as templates and the primers pairs (WKp1 and WKp2) and (WSe1 and WSe2) (Table S1), digested with NdeI/XhoI and cloned into the same sites of pET28MHL. These constructs allow the production of His-tagged FtsW$_{kp}$ and His-tagged FtsW$_{Se}$ respectively.

pCIP1051. The DNA encoding the full-length *E. coli murJ* gene was amplified by PCR from *E. coli* K12 genomic DNA using the MJ1 and MJ2 primers and inserted into the BamHI/HindIII sites of pETDuet-1 to give rise to pCIP1051. This construct allows the production of His-tagged MurJ.

Supplementary figures

Figure S1

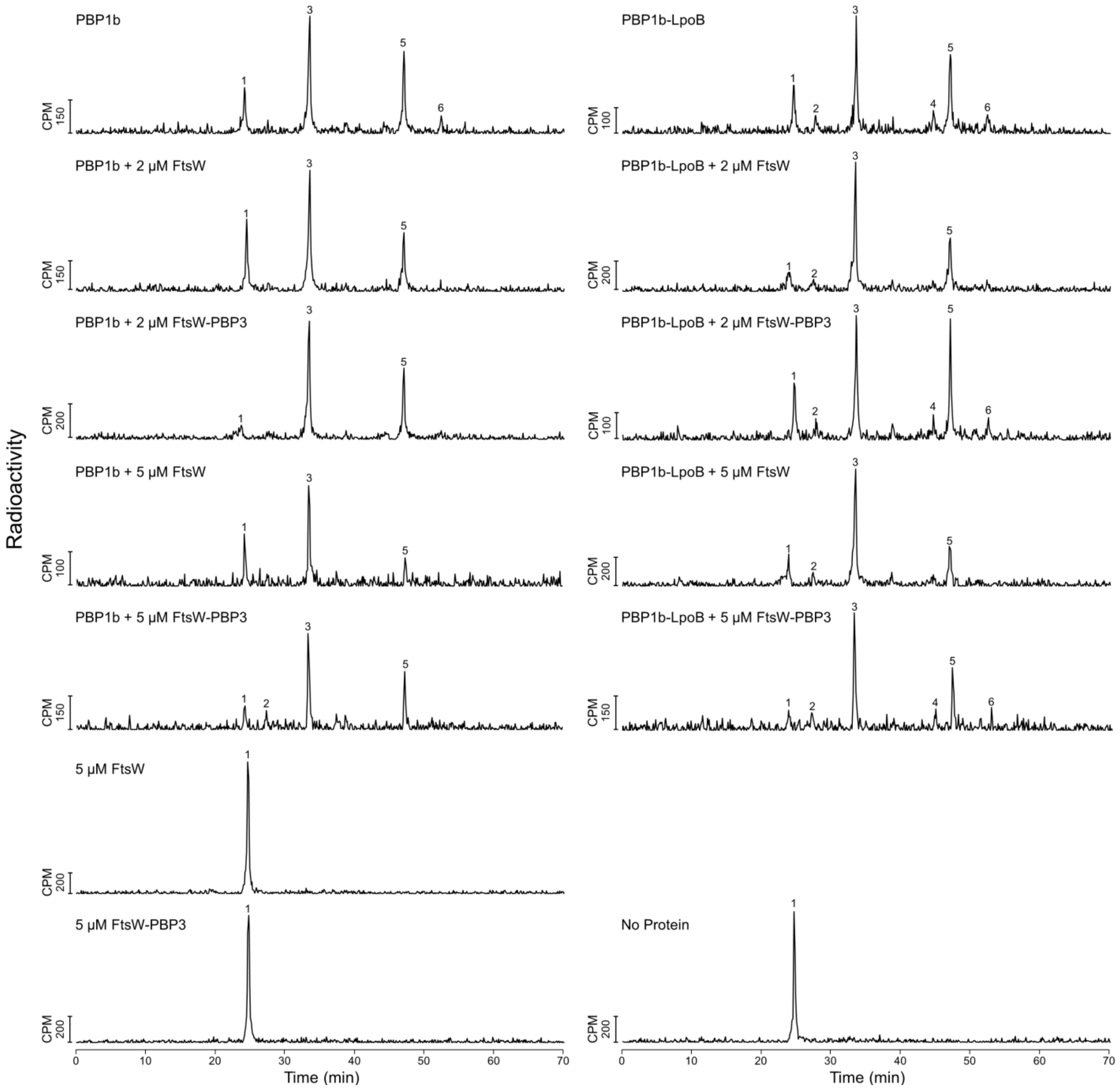

Figure S1. Effect of FtsW and the FtsW-PBP3 complex on the activity of PBP1b *in vitro*. HPLC chromatograms show the muropeptide profile from *in vitro* PG assays: PBP1b or PBP1b-LpoB was incubated with lipid II in the presence or absence of FtsW or FtsW-PBP3. The resulting PG was digested with cellosyl, resulting muropetides were reduced with sodium borohydride and separated by HPLC. Peak 1, monophosphorylated disaccharide pentapeptide; peak 2, disaccharide tetrapeptide; peak 3, disaccharide pentapeptide; peak 4, bis-disaccharide tetratetrapeptide; peak 5, bis-disaccharide tetrapentapeptide; peak 6, tris-disaccharide tetratetrapeptide.

Figure S2

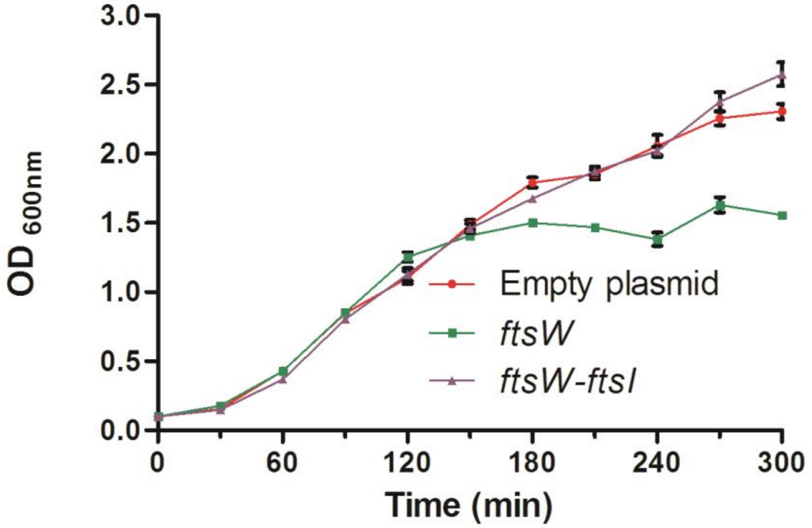

Figure S2. *E. coli* C43 was transformed with empty pET-Duet plasmid, or pET Duet containing *ftsW* gene or both FtsW and *ftsI* genes. The cells were grown in LB medium at 37°C and the optical density (OD) at 600nm of the cell cultures were monitored over time.

Supplementary table S1. Oligonucleotides used in this study.

Primers	Constructs
S510A1: 5'-CGTTCGATTGGTGCGCTTGCAAAACCAG<br>S510A2: 5'-CTGGTTTTGCAAGCGCACCAATCGAACG	pDML924 (S510A)
IW1: 5'-GCCGGATCCGAAAGCAGCGGCGAAAACGCAG<br>IW2: 5'-GCCAAGCTTACGATCTGCCACCTGTCCCCTCG<br>IW3: 5'-GGCAGATCTCCGTTTATCTCTCCCTCGCCTGAAAATG<br>IW4: 5'-CGGCTCGAGTCATCGTGAACCTCGTACAAACGCCTG	pDML2040
WI1: 5'-GCCGGATCCGCGTTTATCTCTCCCTCGCCTGAAAATG<br>WI2: 5'-GCCAAGCTTTCATCGTGAACCTCGTACAAACGCCTG<br>WI3: 5'-GCCAGATCTCAAAGCAGCGGCGAAAACGCAG<br>WI4: 5'-GCCCTCGAGTTACGATCTGCCACCTGTCCCCTCG	pDML2041
Nhis1: 5'-GCCATATGGCACAACGAGATTATGTACGCC<br>Nhis2: 5'-GGCCTCGAGACCCCCGGCGGCGAGCCG	pDML2032
SN1b1: 5'-GAGCCATGGGCATGCCGCGCAAAGGTAAGGGCAAAGG<br>SN1b2: 5'-GACGGATCCTTAATTACTACCAAACATATCCTTGATCCAACCGG	pDML2045
W1b1: 5'-TATACATATGGCCGGGAATGACCGCGAG<br>W1b2: 5'-CAGACTCGAGTTAATTACTACCAAACATATCCTTGATCCAAC	pCIP1000
WHA1: 5'-GCCAGGATCCGCGTTTATCTCTCCCTCGCCTGA<br>WHA2: 5'-CCGCGAATTCTCATCGTGAACCTCGTACAAACGCC	pCIP1083
WKp1: 5'-AGGTTGCATATGCGTTTCTCTC<br>WKp2: 5'-ACCGCCTCGAGTCATCGCACA	pET28MHL-W_{Kp}
WSe1: 5'-CGAAGGAGTTAGGTTGCATATGCGTTTATC<br>WSe2: 5'-GTTGACCACCTCGAGTCATCGAGAA	pET28MHL-W_{Se}
Mj1: 5'-GCCAGGATCCGAATTTATTAAAATCGCTGGCCGCCGTC<br>Mj2: 5'-CCGCAAGCTTTTACACCGTCCGGCGGGCAAATTC	pCIP1051

Supplementary references

1. Terrak, M. *et al.* The catalytic, glycosyl transferase and acyl transferase modules of the cell wall peptidoglycan-polymerizing penicillin-binding protein 1b of Escherichia coli. *Mol Microbiol* **34,** 350–364 (1999).

2. Piette, A. *et al.* Structural determinants required to target penicillin-binding protein 3 to the septum of Escherichia coli. *J Bacteriol* **186,** 6110–7 (2004).

3. Pastoret, S. *et al.* Functional analysis of the cell division protein FtsW of Escherichia coli. *J Bacteriol* **186,** 8370–8379 (2004).

4. Müller, P. *et al.* The Essential Cell Division Protein FtsN Interacts with the Murein (Peptidoglycan) Synthase PBP1B in Escherichia coli. *J Biol Chem* **282,** 36394–36402 (2007).
